# Cascade Hydroxyl Radical-Generating and Ferroptosis-Inducing Nanofiber System for the Therapy of Oral Squamous Cell Carcinoma

**DOI:** 10.3390/molecules29163964

**Published:** 2024-08-22

**Authors:** JiHye Park, Qiaojun Hao, Da In Jeong, Hyun-Jin Kim, Sungyun Kim, Song Yi Lee, Seongnam Chu, Usok Hyun, Hyun-Jong Cho

**Affiliations:** 1 Department of Pharmacy, College of Pharmacy, Kangwon National University, Chuncheon 24341, Republic of Korea; 2 Kangwon Institute of Inclusive Technology, Kangwon National University, Chuncheon 24341, Republic of Korea

**Keywords:** chemodynamic therapy, ferroptosis, nanofiber, oral cancer, starvation therapy

## Abstract

Nanofiber (NF) membrane systems that can provide cascade catalytic reaction and ferroptosis induction were developed for oral cancer therapy. Glucose oxidase (GOx) and aminoferrocene (AF) were introduced into the NF system for glucose deprivation/H_2_O_2_ generation and OH radical generation, respectively. GOx offers starvation therapy and AF (including iron) provides chemodynamic therapy/ferroptosis for combating oral cancer. GOx (water-soluble) and AF (poorly water-soluble) molecules were successfully entrapped in the NF membrane via an electrospinning process. GOx and AF were incorporated into the polyvinyl alcohol (PVA)-based NF, resulting in PVA/GOx/AF NF with fast disintegration and immediate drug-release properties. In oral squamous cell carcinoma (YD-9 cells), the PVA/GOx/AF NF group exhibited higher cytotoxicity, antiproliferation potential, cellular ROS level, apoptosis induction, lipid ROS level, and malondialdehyde level compared to the other NF groups. The electrospun PVA/GOx/AF NF can be directly applied to oral cancer without causing pain, offering starvation/chemodynamic therapy and ferroptosis induction.

## 1. Introduction

For oral cancers, surgery, radiation therapy, and chemotherapy have generally been adopted as primary treatments [1]. While a malignant tumor mass can be directly removed by surgery, this can induce impairments in speaking, breathing, and eating [1,2]. Radiation may reduce the tumor size; however, it has several complications, such as infection, inflammation, nausea, and vomiting [1]. Typical chemotherapeutic drugs, such as carboplatin, cisplatin, docetaxel, hydroxyurea, methotrexate, paclitaxel, and 5-fluorouracil, have been used for oral cancers [3]. Several pharmaceutical formulations, such as gels, nanofibers, and nanoparticles, have been developed, and their anticancer activities have been assessed in oral cancer cell culture and animal models [3].

In addition to conventional chemotherapy, relatively new approaches, including immunotherapy, photodynamic therapy, photothermal therapy, and sonodynamic therapy, have been explored for treating oral cancers [4,5,6,7]. Additionally, reactive oxygen species (ROS)-based therapy using starvation and chemodynamic concepts has been introduced to kill oral cancer cells in combination with novel delivery systems [8]. Starvation therapy is based on the decomposition of highly accumulated glucose in cancer cells via the Warburg effect [9]. In chemodynamic therapy, provided H_2_O_2_ is converted to highly toxic ROS, such as hydroxyl radicals [4]. Combined starvation/chemodynamic therapy has been widely applied to other types of cancers, and delivery systems can enhance anticancer efficacies [10,11].

In our previous study [8], a nanofiber (NF) mat for cascade chemodynamic therapy (using glucose oxidase (GOx) and MnO_2_) and chemotherapy (using rapamycin) for oral cancers was developed. The cascade catalytic reaction, based on GOx (glucose to H_2_O_2_) and MnO_2_ (H_2_O_2_ to OH radical) for chemodynamic therapy, was combined with the inhibition of mammalian target of rapamycin (mTOR) protein kinase (by rapamycin) to combat oral squamous cell carcinoma. In this investigation, aminoferrocene (AF) was selected for OH radical generation (by the Fenton reaction) and ferroptosis induction with the aid of GOx. Ferroptosis is a non-apoptotic regulated cell death mechanism that involves iron-dependent lipid peroxidation, leading to cancer cell death [12]. To the best of our knowledge, the combination of starvation/chemodynamic therapy and ferroptosis against oral cancers using a locoregional approach is being trialed for the first time in this investigation. Apoptosis (based on ROS generation) and ferroptosis (involving iron in ferrocene) can boost the therapeutic efficacies against oral cancers. Moreover, the electrospun polyvinyl alcohol (PVA) NF membrane containing GOx and AF can be quickly disintegrated, allowing the drug cargo to be released immediately upon local application to oral cancers. The developed NF membrane system can be an ideal candidate for starvation/chemodynamic therapy and ferroptosis induction in oral squamous cell carcinoma.

## 2. Results and Discussion

### 2.1. Fabrication and Physicochemical Evaluation of NF Systems

**Figure 1:**
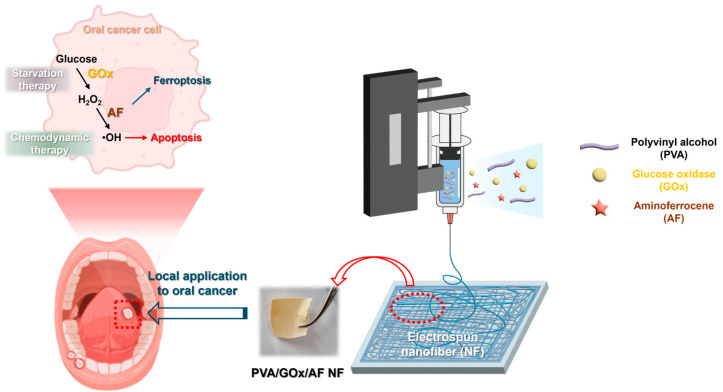
Schematic of PVA/GOx/AF NF for local oral cancer therapy.

Electrospun NF systems were fabricated for local delivery of GOx and AF to oral cancer for starvation/chemodynamic therapy and induction of ferroptosis (Figure 1). Aqueous-soluble GOx and poorly water-soluble AF (at current concentrations) were homogeneously entrapped within the NF structure, and the PVA-based NF membrane exhibited rapid wetting, disintegration, and dissolution properties. The NF membrane was transformed to a nanoparticle (NP) structure upon dispersing in aqueous media, and it may be internalized in oral cancer cells.

**Figure 2:**
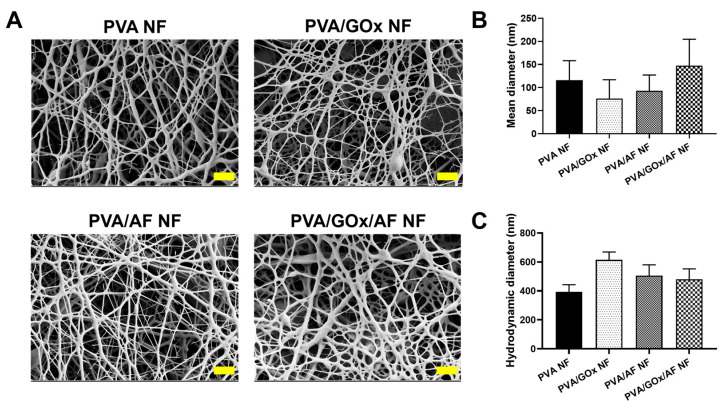
Physicochemical properties of NF systems. (**A**) FE-SEM images of PVA NF, PVA/GOx NF, PVA/AF NF, and PVA/GOx/AF NF. Scale bar: 1 μm. (**B**) Mean diameter of NF read from FE-SEM images. Each point represents mean ± SD (*n* ≥ 3). (**C**) Hydrodynamic size of NF dispersion. Each point represents mean ± SD (*n* = 3).

The electrospinning process of PVA-based NF membranes for immediate disintegration and dissolution was previously optimized in our studies [8,13,14]. PVA and GOx were dispersed in distilled water (DW), and AF was solubilized in ethanol. This mixture was used as the working solution for electrospinning. PVA NF, PVA/GOx NF, PVA/AF NF, and PVA/GOx/AF NF were fabricated using the electrospinning process, and their morphological shapes were observed by field emission scanning electron microscope (FE-SEM) imaging (Figure 2A). A nanoweb structure was seen in all NF systems, indicating proper manufacturing conditions. The mean diameter of the fibers was measured from the FE-SEM image (Figure 2B), with average values ranging from 76 nm to 147 nm. Notably, the mean diameter of PVA/GOx/AF NF was 147 nm, representing the characteristic size of the NF structure. The relative enzyme activity value of GOx in PVA/GOx/AF NF was approximately 100%, indicating the proper maintenance of GOx’s biological function embedded in the NF structure. The encapsulation efficiency values of AF in PVA/AF NF and PVA/GOx/AF NF were 72% and 76%, respectively. The hydrodynamic size of NF dispersion was also measured, as shown in Figure 2C. These values for PVA NF, PVA/GOx NF, PVA/AF NF, and PVA/GOx/AF NF were 393 nm, 614 nm, 505 nm, and 481 nm, respectively. NF may be internalized into the cancer cells as NP structure.

**Figure 3:**
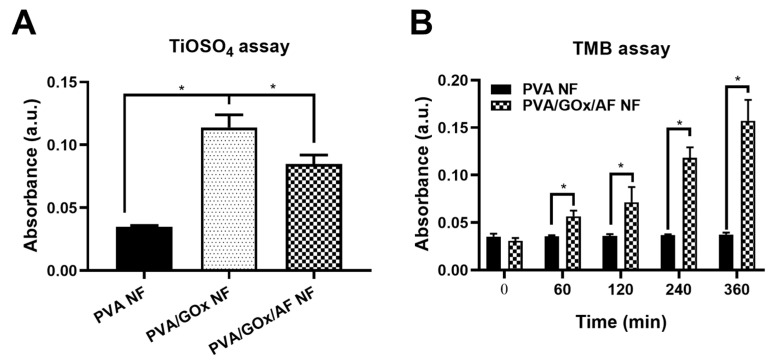
Catalytic features of NF systems. (**A**) TiOSO_4_ assay data suggesting H_2_O_2_ generation. Each point represents mean ± SD (*n* = 3). * *p* < 0.05, between indicated groups. (**B**) TMB assay data indicating OH radical production. Each point represents mean ± SD (*n* = 4). * *p* < 0.05, between indicated groups.

Cascade catalytic functions of the GOx/AF-combined NF system were investigated using titanium(IV) oxysulfate (TiOSO_4_) and 3,3′,5,5′-tetramethylbenzidine (TMB) assays (Figure 3A,B). TiOSO_4_ is used to identify H_2_O_2_ based on a colorimetric method [15]. As shown in Figure 3A, there was a significant increase in the PVA/GOx NF group compared to the PVA NF group, primarily due to the generation of H_2_O_2_ from the enzymatic reaction between GOx and glucose. This glucose decomposition may provide starvation therapy in cancer cells that have higher glucose levels based on the Warburg effect [16]. Interestingly, the absorbance value of the PVA/GOx/AF NF group was lower than that of the PVA/GOx NF group, meaning there was appropriate decomposition of H_2_O_2_ to OH radicals by ferrocene (in AF). The generation of OH radicals by the GOx/AF combination can provide a chemodynamic therapy option against oral cancer. These data suggest the successful cascade reaction from glucose to OH radicals facilitated by the GOx/AF combination installed in the NF system. The OH radical generation potential of the fabricated PVA/GOx/AF NF system was further investigated with the TMB assay (Figure 3B). OH radicals oxidized TMB (colorless) to a charge-transfer complex (blue), allowing the assessment of OH radical production by detecting absorbance values [17]. Compared to the PVA NF group, the PVA/GOx/AF group showed higher absorbance values at 60–360 min, indicating that glucose was decomposed to H_2_O_2_ by GOx and that H_2_O_2_ was further transformed to OH radicals by AF in the PVA/GOx/AF NF system. These TMB assay data also support the successful cascade catalytic reaction of GOx/AF installed in the NF system for generating OH radicals (targeting chemodynamic therapy), consistent with the TiOSO_4_ assay data (Figure 3A).

### 2.2. Disintegration, Wetting, and Dissolution Characteristics

**Figure 4:**
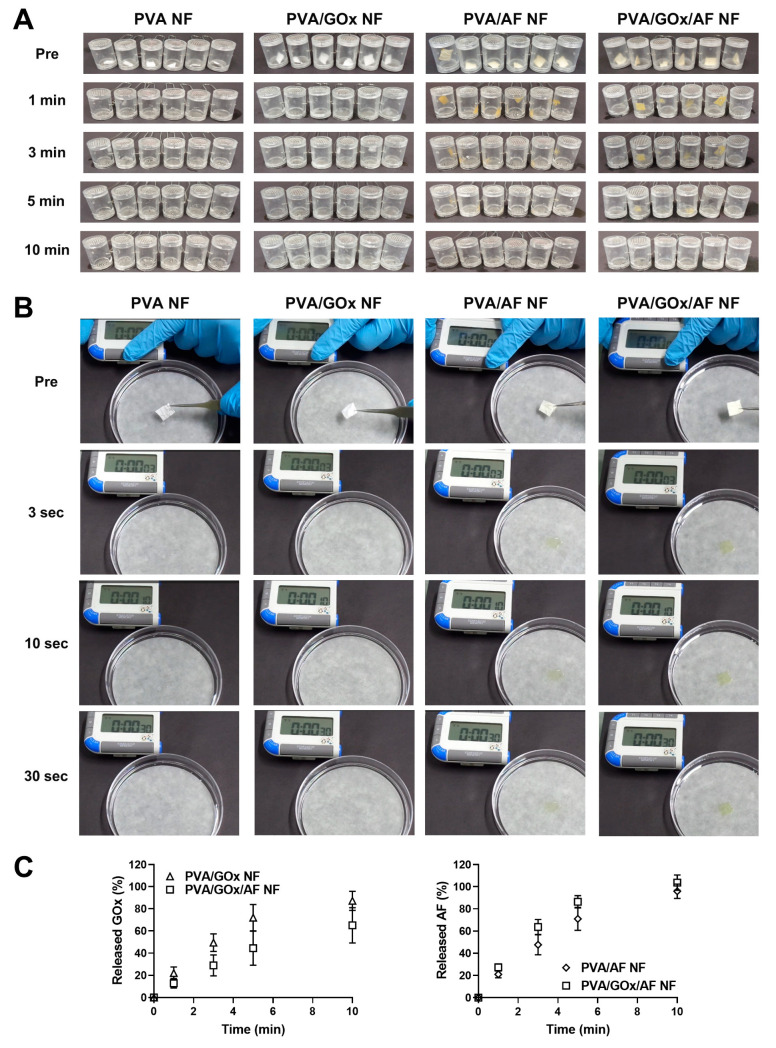
Disintegration, wetting, and dissolution features of NF systems. Disintegration (**A**) and wetting (**B**) images of NF systems are shown. (**C**) GOx and AF release profiles of NF systems. Each point represents mean ± SD (*n* = 3).

The quick disintegration and immediate drug dissolution properties of the designed NF systems were tested (Figure 4). In our previous studies [8,13,14], PVA-based NF membranes exhibited fast disintegration and drug-release characteristics. In this study, the disappearance of the PVA NF membrane was shown at 1 min, with almost complete disintegration evident by 10 min (Figure 4A). In the case of the PVA/GOx NF group, no membrane fragments were seen at 10 min. While some NF membrane fragments remained at 3 min in the PVA/AF NF and PVA/GOx/AF NF groups, no obvious specimens were present at 10 min, implying complete disintegration.

The wetting feature of the electrospun PVA-based NF system was also explored (Figure 4B). Disintegration and drug dissolution may begin with the surface wetting of the NF membrane and the intrusion of water molecules. All NF groups exhibited complete wetting, indicated by the disappearance of the NF membrane, within 3 s. This means that the fabricated electrospun NF membrane may adhere promptly and be internalized into the cancer cells.

Drug dissolution properties of the designed NF systems were also tested at pH 6.8 (Figure 4C). The released amounts of GOx from PVA/GOx NF and PVA/GOx/AF NF were 87% and 65%, respectively, within 10 min. The release amounts of AF from PVA/AF NF and PVA/GOx/AF NF reached 100% at 10 min, indicating immediate release of AF despite its poor aqueous solubility. These quick-release profiles of GOx and AF within 10 min suggest instant endocytosis of the drug cargo in the oral cavity.

### 2.3. In Vitro Anticancer Activities

**Figure 5:**
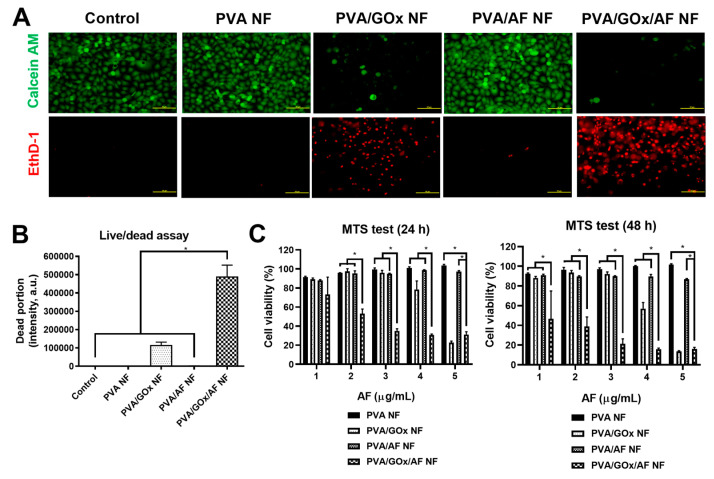
Cytotoxic activity tests of NF systems in YD-9 cells. (**A**) Live/dead assay data. Calcein AM and EthD-1 images (by fluorescence microscopy) are shown. Scale bar length: 50 μm. (**B**) Dead portion data are also plotted. Each point represents mean ± SD (*n* ≥ 3). * *p* < 0.05 between indicated groups. (**C**) MTS test data. Cell viability values following 24 and 48 h incubation are plotted. Each point represents mean ± SD (*n* ≥ 3). * *p* < 0.05 between indicated groups.

**Figure 6:**
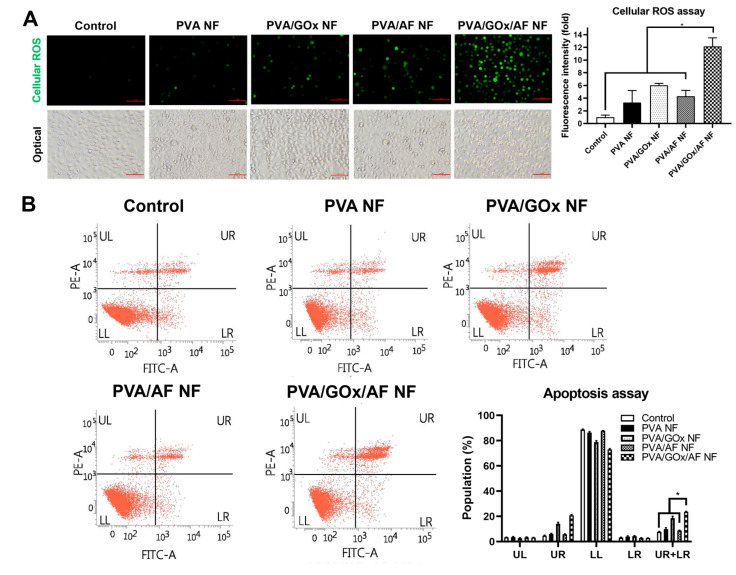
Investigation of cytotoxic mechanisms of NF systems in YD-9 cells. (**A**) Cellular ROS test data. Fluorescence (for cellular ROS degree) and optical images are shown. Scale bar length: 100 μm. Fluorescence intensity ratios (fold increase) are plotted. Each point represents mean ± SD (*n* ≥ 3). * *p* < 0.05 between indicated groups. (**B**) Apoptosis assay data. Population percentage values are plotted. Each point represents mean ± SD (*n* ≥ 3). * *p* < 0.05 between indicated groups.

**Figure 7:**
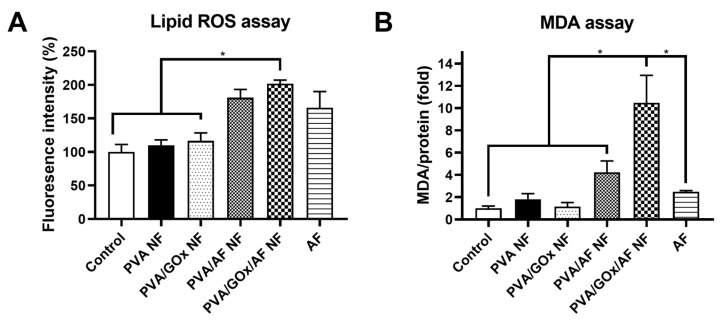
Ferroptosis induction tests in YD-9 cells. (**A**) Lipid ROS assay data. Fluorescence intensity ratios (%) are plotted. Each point represents mean ± SD (*n* ≥ 3). * *p* < 0.05 between indicated groups. (**B**) MDA assay data. Relative MDA levels normalized by protein contents (fold) are plotted. Each point represents mean ± SD (*n* ≥ 3). * *p* < 0.05 between indicated groups.

Anticancer activities of GOx/AF-combined NF systems were demonstrated in YD-9 cells (Figure 5, Figure 6 and Figure 7). The cytotoxic potential of the GOx/AF-embedded NF system was explored using a live/dead assay (Figure 5A,B). Green (calcein AM) and red (ethidium homodimer-1 (EthD-1)) fluorescence signals indicate live and dead cells, respectively. Under the current treatment conditions, PVA/GOx NF showed higher efficacy in inducing cancer cell death compared to PVA/AF NF. PVA NF did not induce significant cell death, suggesting that the cytotoxic effects are primarily due to GOx and AF. The PVA/GOx/AF NF group had a significantly higher proportion of dead cells compared to the other groups (*p* < 0.05).

The antiproliferation potential of the designed NF system was explored in YD-9 cells using 3-(4,5-dimethylthiazol-2-yl)-5-(3-carboxymethoxyphenyl)-2-(4-sulfophenyl)-2H-tetrazolium (MTS)-based assay (Figure 5C). At 24 and 48 h, the PVA/GOx/AF NF group had lower cell viability values compared to the PVA NF, PVA/GOx NF, and PVA/AF NF groups at 2–4 μg/mL AF concentrations (*p* < 0.05). At 5 μg/mL AF concentration, the PVA/GOx/AF NF group had similar cell viability values to the PVA/GOx NF group; however, both were lower than the PVA NF and PVA/AF NF groups. These data suggest that the fabricated electrospun PVA/GOx/AF NF has superior antiproliferation potential based on apoptosis and ferroptosis induction effects with cascade catalytic reactions.

The efficacy of cellular ROS generation by the GOx/AF-entrapped NF system was investigated in YD-9 cells (Figure 6A). The PVA NF group showed negligible ROS level, meaning no ROS generation effect. Interestingly, the PVA/GOx/AF NF group exhibited higher ROS level compared to the PVA/GOx NF and PVA/AF NF groups (*p* < 0.05), likely due to synergistic mechanisms. This can be attributed to the successful cascade catalytic reactions of GOx (from glucose to H_2_O_2_) and AF (from H_2_O_2_ to OH radical).

The apoptosis induction efficiency of the GOx/AF-incorporated NF system was studied in YD-9 cells via Annexin V and propidium iodide (PI) staining (Figure 6B). The sum of the population data in the upper right (UR) and lower right (LR) panels indicates the apoptosis rates (in early and late phases). It appears that the PVA/GOx NF group had a higher mean value of the (UR + LR) panel population compared to the PVA/AF NF group. Importantly, the value of the PVA/GOx/AF NF group was obviously higher than those of the other groups (*p* < 0.05), suggesting superior apoptosis induction effects.

The lipid ROS level following the application of the designed NF system was analyzed in YD-9 cells (Figure 7A). Cellular ROS is known to be involved in the oxidation of lipids, and lipid peroxidation of phospholipid bilayers may result in ferroptosis [18]. Iron included in AF can induce lipid ROS in cancer cells, and this mechanism was confirmed in this study [19]. The mean values of the PVA/AF NF and AF groups were higher than those of the control, PVA NF, and PVA/GOx NF groups. Interestingly, the lipid ROS level of the PVA/GOx/AF NF group was also obviously higher than those of control, PVA NF, and PVA/GOx NF groups (*p* < 0.05). This implies that the designed PVA/GOx/AF NF system can induce ferroptosis in cancer cells [19].

The ferroptosis induction effect of the electrospun NF system was further investigated by measuring malondialdehyde (MDA) levels in YD-9 cells (Figure 7B). MDA is one of the lipid peroxidation products; therefore, it can be used as a ferroptosis indicator. The PVA/GOx/AF NF group had a higher MDA level than the other groups, implying the efficient cellular entry of AF entrapped in PVA/GOx/AF NF as an NP structure and the most efficient ferroptosis induction effect. MDA, 4-hydroxy-nonenal (4-HNE), and F2-isoprostane 15(S)-8-iso-prostaglandin F2α (15(S)-8-iso-PGF2α) have been regarded as representative products of lipid peroxidation, and those three substances are produced from polyunsaturated fatty acids by chemical and enzyme reactions [20]. Lipid ROS level may indicate total ROS content, which can be measured by C11-BODIPY581/591 [21], while MDA is one of several lipid peroxidation products. Therefore, different data between those lipid ROS and MDA levels (in Figure 7A,B) imply different contributions of other ROS products. The designed GOx and AF-entrapped NF system could be conveniently and painlessly applied to the local therapy of oral cancers.

## 3. Materials and Methods

### 3.1. Materials

AF, D-glucose, GOx (from *Aspergillus niger*), PVA (molecular weight: 30−70 kDa), TiOSO_4_ solution, 2-thiobarbituric acid, 2′,7′-dichlorofluorescin diacetate (DCFH-DA), and 3,3′,5,5′-tetramethylbenzidine dihydrochloride hydrate (TMB DH) were provided by Sigma–Aldrich (Saint Louis, MO, USA). Fetal bovine serum (FBS), phosphate-buffered saline (PBS), and RPMI 1640 (including 300 mg/L L-glutamine and 25 mM NaHCO_3_) cell culture medium were acquired from Welgene Inc. (Gyeongsan, Republic of Korea). Penicillin/streptomycin was purchased from Gibco Life Technologies, Inc. (Grand Island, NY, USA).

### 3.2. Preparation of NF System and Their Physicochemical Assessments

The fast-dissolving PVA-based NF system was fabricated using a reported method [8,13,14]. For the preparation of PVA/GOx/AF NF, PVA (4 g) was dissolved in DW (15 g) by heating and then cooled to room temperature. GOx (2 mg) was dissolved in DW (1 g) and mixed with PVA dispersion. AF (4 mg) was solubilized in ethanol (2 g) and blended with PVA/GOx mixture at a 1:1 weight ratio to make a working solution. This working solution was filled into a syringe connected with a stainless-steel needle (25 G). The voltage (25 kV), distance from needle tip to collector (15 cm), and flow rate (1 mL/h) were established for the fabrication of NF. PVA NF, PVA/GOx NF, and PVA/AF NF were prepared with the same composition and apparatus conditions, omitting GOx/AF, AF, and GOx, respectively.

The shape and diameter of the NF were measured with a variable pressure-field emission scanning electron microscope (VP-FE-SEM; SUPRA 55VP, Carl Zeiss, Oberkochen, Germany). NF specimens were coated with Au, and SEM images were acquired.

The bioactivity of GOx in NF was measured using the Amplex Red Glucose/Glucose Assay Kit (Invitrogen, Waltham, MA, USA). GOx and PVA/GOx/AF NF (at 2 μg/mL of GOx) were dissolved in PBS and treated with the assay kit. Then, an aliquot (50 μL) of each sample was transferred to a 96-well plate, and the absorbance at 570 nm was measured with a microplate reader (SpectraMax i3, Molecular Devices, Sunnyvale, CA, USA).

The entrapment efficiency values of AF in the NF specimens were analyzed by high-performance liquid chromatography (HPLC; Agilent 1260 Infinity II, Agilent Technologies, Santa Clara, CA, USA). A reverse phase C18 column (Kinetex, 150 mm × 4.6 mm, 2.6 μm; Phenomenex, Torrance, CA, USA) was connected to the HPLC system, and the mobile phase consisting of 0.1% trifluoroacetic acid in DW and acetonitrile (9:1, *v*/*v*) was flowed at 1 mL/min. The sample volume was 20 μL, and the absorbance was detected at 415 nm with the HPLC system.

Hydrodynamic size data of the NF system dispersed in DW were obtained using the dynamic light scattering method (ELS-Z1000; Otsuka Electronics, Tokyo, Japan). PVA NF, PVA/GOx NF, PVA/AF NF, and PVA/GOx/AF NF (10 mg) were dispersed in DW and vortex-mixed for 2 min. Particle size values of the NF dispersions were then analyzed.

The generation efficiency of H_2_O_2_ from glucose with the designed NF systems was evaluated using the TiOSO_4_ assay [8]. An aliquot (0.2 mL) of PVA NF, PVA/GOx NF, and PVA/GOx/AF NF (at 1 mg/mL of AF) was placed into a dialysis tube (molecular weight cut-off (MWCO): 12–14 kDa) and transferred to a disposable plastic tube. An aliquot (2 mL) of DW or D-glucose in DW (100 mM) was added to that tube and incubated for 60 min at 37 °C. A determined volume (2 mL) of TiOSO_4_ solution (30 mM in DW) was added to the tube and incubated for a further 30 min. Each sample was transferred to a 96-well plate, and the absorbance at 450 nm was measured with a microplate reader.

The efficacy of OH radical generation from H_2_O_2_ was assessed using the TMB assay [8]. A dispersion (0.5 mL) of PVA NF or PVA/GOx/AF NF (at 25 mg/mL) was added to a dialysis bag (MWCO: 12–14 kDa) and transferred to a conical tube. D-glucose solution (10 mM in DW, 2.5 mL) was added to the tube and incubated at 37 °C for 60 min. TMB DH solution (10 mM in DW, 2.5 mL) was then added and incubated at 37 °C. The media were collected at 0, 60, 120, 240, and 360 min, and the absorbance at 650 nm was measured with a microplate reader.

### 3.3. Disintegration, Wetting, and Dissolution Studies

The disintegration behaviors of the designed electrospun NF systems were investigated using a disintegration tester (TD-20S, Campbell Electronics, Mumbai, India) [8]. PVA NF, PVA/GOx NF, PVA/AF NF, and PVA/GOx/AF NF (1 cm × 1 cm) were placed in the basket and immersed in PBS (pH 6.8) at 37 ± 2 °C for 10 min with up-and-down movement. Images were obtained at pre, 1, 3, 5, and 10 min.

The wetting property of the electrospun NF system was tested using the reported method [8]. Filter paper (110 mm of diameter; CHMLAB group, Barcelona, Spain) on a Petri dish (diameter: 150 mm) was soaked with PBS (pH 6.8). PVA NF, PVA/GOx NF, PVA/AF NF, and PVA/GOx/AF NF (1 cm × 1 cm) were placed onto the filter paper, and the images were acquired at pre, 3, 10, and 30 s.

Release profiles of GOx from electrospun NF systems were tested at pH 6.8 and 37 °C. PVA/GOx NF and PVA/GOx/AF NF, containing 2 μg of GOx, were added to 10 mL of PBS (pH 6.8). An aliquot (0.2 mL) of release medium was taken at 1, 3, 5, and 10 min. The GOx concentration was measured using a glucose oxidase activity assay kit (Abcam, Cambridge, UK), and the complete dispersion of the NF formulation was used to calculate relative release ratios (%).

Release patterns of AF from electrospun NF systems were investigated at pH 6.8. PVA/AF NF and PVA/GOx/AF NF (containing 1 mg of AF) were immersed in 5 mL of PBS (pH 6.8) at 37 °C and 100 rpm. An aliquot (0.2 mL) of the release medium was collected at 1, 3, 5, and 10 min. The concentrations of AF in the release media were determined by measuring the absorbance values at 415 nm with a microplate reader.

### 3.4. In Vitro Anticancer Activity Assays

The cytotoxic potential of the designed NF system was investigated using a live/dead assay in YD-9 cells. YD-9 cells (squamous cell carcinoma from human buccal tissue) were acquired from the Korean Cell Line Bank (Seoul, Republic of Korea). The cells were cultured in RPMI 1640 including FBS (10%, *v*/*v*) and penicillin/streptomycin (1%, *v*/*v*) at 5% CO_2_ and 37 °C. Cells were seeded onto a 24-well plate at a density of 2 × 10^5^ cells/well and incubated overnight. Subsequently, PVA NF, PVA/GOx NF, PVA/AF NF, and PVA/GOx/AF NF (containing 1.5 μg/mL of AF) were added to the cells and incubated for 6 h. The Live/Dead^®^ Viability/Cytotoxicity Kit (Thermo Fisher Scientific, Waltham, MA, USA) was used to stain live cells (calcein AM, green) and dead cells (EthD-1, red). The staining process followed the manufacturer’s protocols, and images were captured using fluorescence microscopy (Eclipse Ts2, Nikon Corp., Tokyo, Japan).

The antiproliferative capability of the electrospun NF system was investigated in YD-9 cells using a colorimetric assay. YD-9 cells were seeded onto a 96-well plate at 5.0 × 10^3^ cells per well and cultured overnight. PVA NF, PVA/GOx NF, PVA/AF NF, and PVA/GOx/AF NF (containing 1, 2, 3, 4, and 5 μg/mL of AF) were applied to the cells and incubated for 24 and 48 h. After removing the NF specimens, CellTiter 96^®^ AQueous One Solution Cell Proliferation Assay Reagent (Promega Corp., Fitchburg, WI, USA) was added to the cells according to the manufacturer’s instructions. The absorbance values at 490 nm were measured using a microplate reader.

Cellular ROS levels following the application of the NF system were detected by a fluorescence indicator. YD-9 cells were seeded in a 24-well plate at 2.5 × 10^5^ cells per well and incubated overnight. PVA NF, PVA/GOx NF, PVA/AF NF, and PVA/GOx/AF NF (containing 4 μg/mL of AF) were applied to the cells and incubated for 6 h. After washing with PBS three times, carboxy-H_2_DCFDA (Thermo Fisher Scientific, 10 μM) was added to the cells and incubated for 30 min. Cellular fluorescence signals were quantified using fluorescence microscopy.

The apoptosis-inducing effects of the designed NF systems were explored in YD-9 cells using the Annexin V and PI staining method. Cells were seeded in a 12-well plate at 5.0 × 10^5^ cells per well and cultured overnight. PVA NF, PVA/GOx NF, PVA/AF NF, and PVA/GOx/AF NF (containing 2 μg/mL of AF) were applied to the cells and incubated for 24 h. After eliminating the NF samples, cells were rinsed with PBS and incubated with FITC Annexin V Apoptosis Detection Kit (BD Pharmingen, BD Biosciences, San Jose, CA, USA) reagents. The cellular fluorescence signals were analyzed using a flow cytometer (FACSverse instrument; BD Bioscience).

The lipid peroxidation-inducing actions of the designed NF systems were investigated in YD-9 cells. Cells were seeded in a 12-well plate at 5.0 × 10^5^ cells per well and incubated overnight. PVA NF, PVA/GOx NF, PVA/AF NF, PVA/GOx/AF NF, and AF (at 10 μg/mL of AF) were applied to the cells and incubated for 6 h. Then, C11-BODIPY581/591 (Molecular Probes, Inc., Eugene, OR, USA; 10 μM) was applied to the cells and incubated for 30 min. The cells were washed twice with PBS, and the cell pellet was collected by centrifugation. After suspending in PBS containing 2% FBS, the cellular fluorescence intensity was quantitatively determined using a flow cytometer.

The ferroptosis induction effect of the designed NF system was evaluated in YD-9 cells by measuring MDA levels. Cells were seeded in a 6-well plate at 5.0 × 10^5^ cells per well and incubated overnight. Cell culture media (1.5 mL) including PVA NF, PVA/GOx NF, PVA/AF NF, PVA/GOx/AF NF, and AF (at 0.2 mg/mL AF) were added to the cells and incubated for 24 h. Cells were washed and lysed, and then specimens were acquired by centrifugation. Cell-lysed specimen (0.08 mL), 0.1% phosphoric acid solution (0.4 mL), and 0.6% 2-thiobarbituric acid solution (0.1 mL) were vortex-mixed and heated at 90 °C for 1 h. The fluorescence intensity was measured at 532 nm (excitation) and 553 nm (emission) using a microplate reader. The protein content of cell-lysed specimens was determined by the BCA assay (Pierce BCA Protein Assay Kit, Thermo Fisher Scientific), and MDA levels were normalized by those values.

### 3.5. Statistical Analysis

Each assay was performed at least three times. All data are presented as the mean ± standard deviation (SD). Statistical analyses were conducted using the two-tailed *t*-test and analysis of variance with post hoc testing.

## 4. Conclusions

The GOx and AF-entrapped NF membrane system was fabricated using the electrospinning method, and its potential for starvation/chemodynamic therapy and ferroptosis induction in oral cancer cells was evaluated. GOx (for glucose decomposition and H_2_O_2_ generation) and AF (for the conversion of H_2_O_2_ to OH radicals) were homogeneously incorporated into the PVA-based NF membrane. The OH radical generation potential of PVA/GOx/AF NF was successfully verified, and the NF membrane system exhibited rapid disintegration and drug dissolution properties within 10 min. In oral squamous cancer cells (YD-9 cells), higher cytotoxicity, antiproliferation capability, ROS levels, apoptosis induction, lipid ROS levels, and MDA levels were observed in the PVA/GOx/AF NF group. The designed NF membrane system can be directly applied to oral cancers without causing pain, and it may exert superior anticancer effects through starvation/chemodynamic therapy and ferroptosis.

## Data Availability

Data are contained within the article.
